# Supplementary material for: Frog-killing chytrid fungi deploy different strategies to regulate intracellular pressure in cell types that have or lack a cell wall
Source: bioRxiv. 2025 May 14:2025.05.13.653819. Preprint. [Version 1] doi: 10.1101/2025.05.13.653819 (PMC12132441; doi:10.1101/2025.05.13.653819)
Supplement: 9 [file NIHPP2025.05.13.653819v1-supplement-9.pdf]

bioRxiv preprint doi: https://doi.org/10.1101/2025.05.13.653819; this version posted May 14, 2025. The copyright holder for this preprint (which was not certified by peer review) is the author/funder, who has granted bioRxiv a license to display the preprint in perpetuity. It is made available under aCC-BY 4.0 International license.

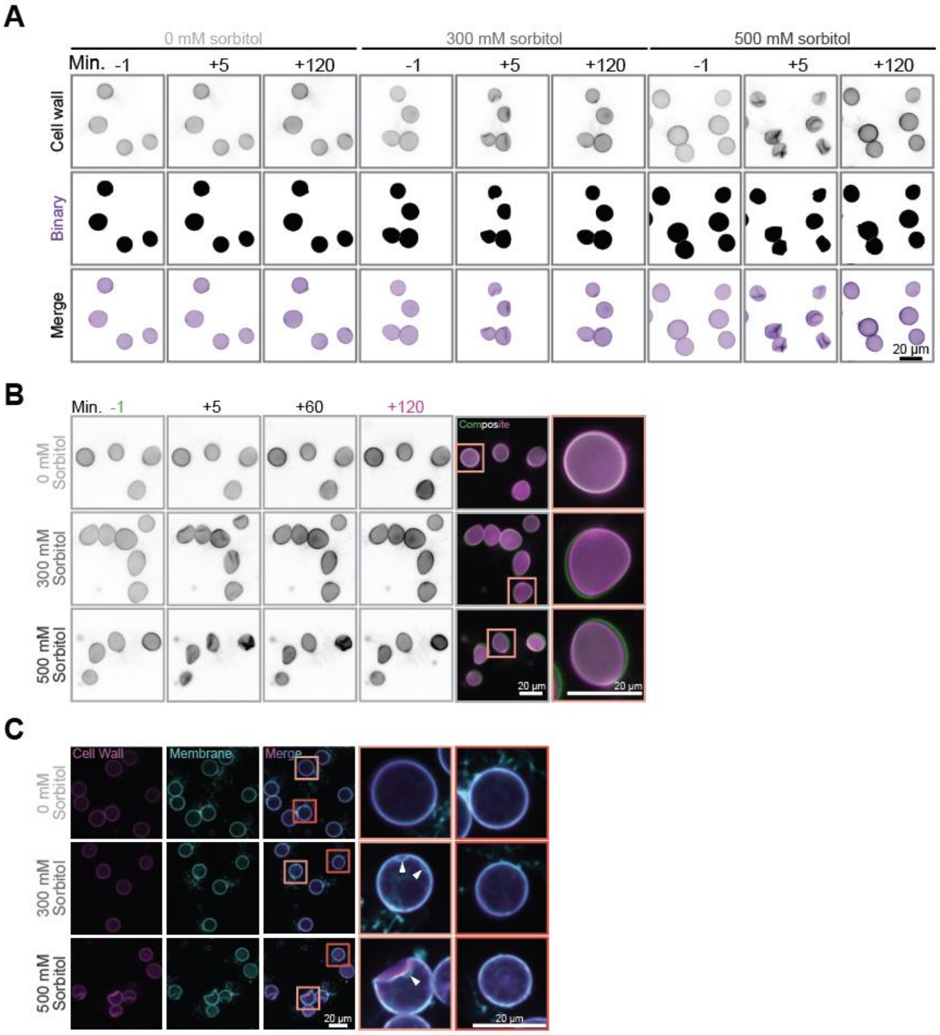

Figure S1. Analysis of *Bd* sporangia responding to hyperosmotic shock. (A) Representative images of *Bd* sporangia cell walls stained with Evans Blue (top) one minute before (-1) and five (+5) and 120 (+120) minutes after treatment with media supplemented with the indicated sorbitol concentration. All cell wall images are adjusted to the same brightness and contrast. Cells were segmented using the cell wall signal in NIS elements (v6.02.03), resulting in a binary layer (purple) that encompasses the cell body **(B)** Representative images of *Bd* sporangia cell walls stained with Evans Blue one minute before (-1) and five (+5), 60 (+60) and 120 (+120) minutes after treatment with media supplemented with the indicated concentration of sorbitol. Composite images show cells one minute before (green) and 120 minutes after (magenta) sorbitol treatment. All images are adjusted to the same brightness and contrast. **(C)** Example images of *Bd* sporangia stained for the cell wall (magenta) and membrane (cyan) after five minutes of treatment with media supplemented with the given sorbitol concentration. Merged images show the overlay of membrane and cell wall. For the 300 and 500 mM sorbitol treatments, insets give one example of a cell with delamination and another example of a cell without delamination from the same field of view. White arrowheads indicate membrane delaminations. All images for each stain are adjusted to the same brightness and contrast.

bioRxiv preprint doi: https://doi.org/10.1101/2025.05.13.653819; this version posted May 14, 2025. The copyright holder for this preprint (which was not certified by peer review) is the author/funder, who has granted bioRxiv a license to display the preprint in perpetuity. It is made available under aCC-BY 4.0 International license.

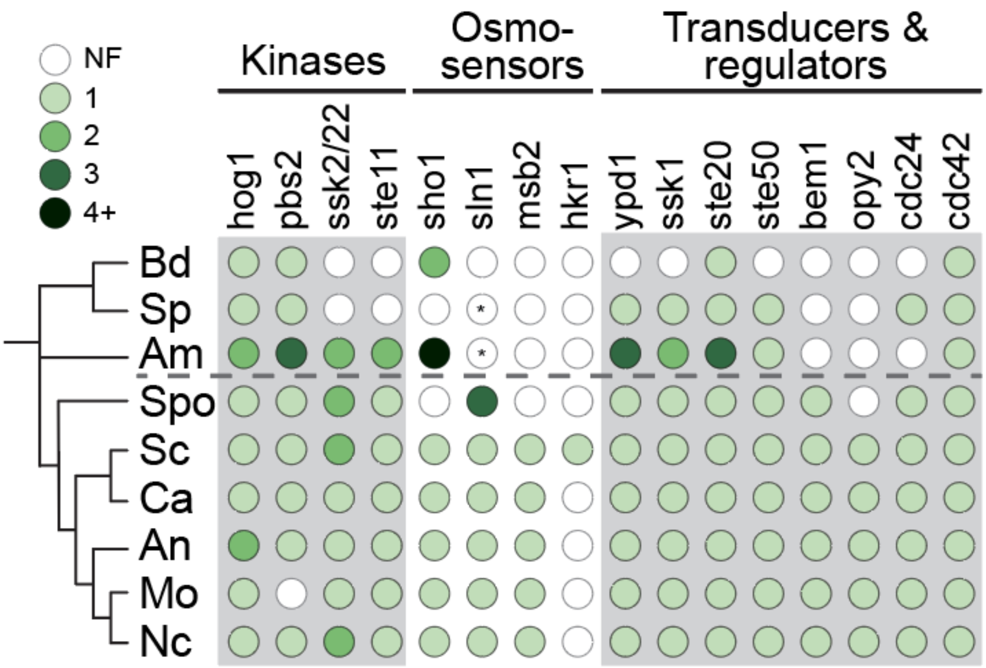

Figure S2. *Bd* and other chytrids have putative homologs of several key components of the HOG pathway. The distribution of proteins known to function in the high osmolarity glycerol pathway (HOG) across fungal taxa. The HOG pathway is the primary pathway used by budding yeast to regulate turgor pressure and has three main classes of proteins: 1) a mitogen activated kinase (MAPK) cascade ending with the MAPK Hog1; 2) osmo-sensors to sense changes in external osmolarity; and 3) transducers and regulators that connect the MAPKs and osmosensors. White-filled circles indicate that homologs are not found (NF), color-filled circles indicate the detection of one or more homologs. Kinase copy numbers were obtained from.[69] Dashed line separates chytrid species (above) from Dikaryotic species (below). *: There is no clear homolog for sln1, but there are putative histidine kinases with predicted transmembrane domains in the given species' genomes. Transmembrane domains are a hallmark of sln1-related histidine kinases.[88,89] *Am*, *Allomyces macrogynus*; *An*, *Aspergillus nidulans*; *Bd*, *Batrachochytrium dendrobatidis*; *Ca*, *Candida albicans*; *Mo*, *Magnaporthe oryzae*; *Nc*, *Neurospora crassa*; *Sc*, *Saccharomyces cerevisiae*; *Sp*, *Spizellomyces punctatus*; *Spo*, *Schizosaccharomyces pombe.*

bioRxiv preprint doi: https://doi.org/10.1101/2025.05.13.653819; this version posted May 14, 2025. The copyright holder for this preprint (which was not certified by peer review) is the author/funder, who has granted bioRxiv a license to display the preprint in perpetuity. It is made available under aCC-BY 4.0 International license.

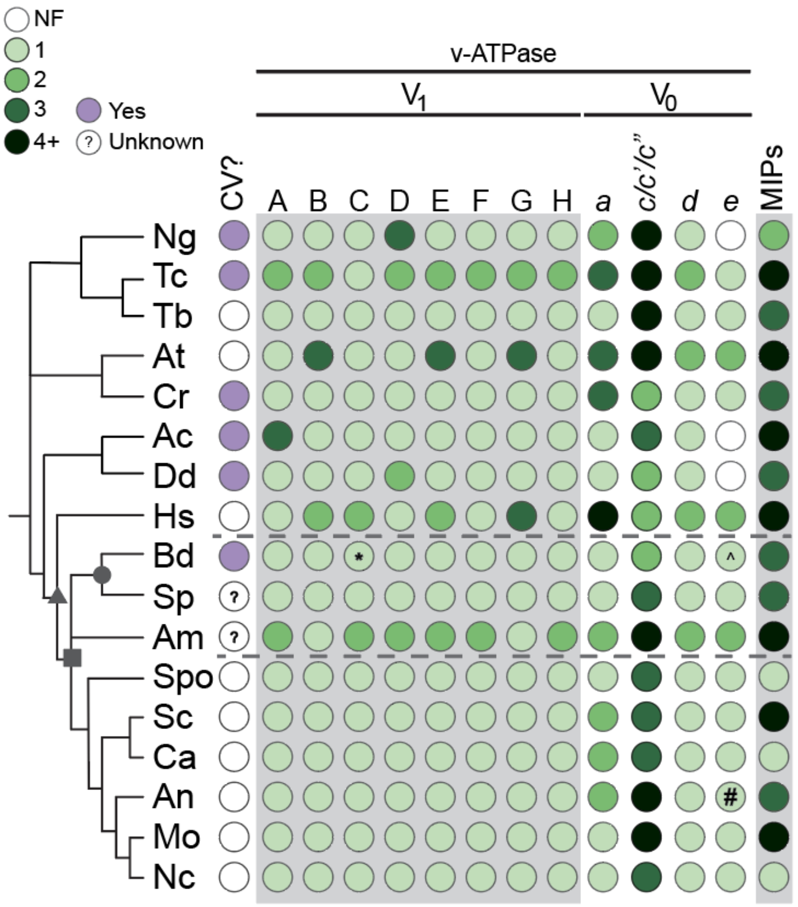

Figure S3. *Bd* and other chytrids have putative aquaporin and vacuolar-ATPase homologs. The distribution of major intrinsic family proteins (MIPs), subunits of the vacuolar-ATPase (v-ATPase), and presence of contractile vacuoles (CV) across taxa. Aquaporins are part of the MIP family of proteins. The v-ATPase is made up of two complexes (V_1 and V_0), each comprising several subunits.[48] White-filled circles indicate that homologs are not found (NF), color-filled circles indicate the detection of one or more homologs. Purple circles indicate the presence of documented CVs in the given organism. *: Homolog is predicted to be in the ubiquitin activating enzyme family (IPR018075), but this likely represents two separate genes erroneously annotated as one. ^: Homolog only identifiable when using the *Manduca sexta* protein (NCBI RefSeq XP_037299296.1) as a query for BLASTp or tBLASTn. #: Homolog is not annotated in the reference genome, and is only identifiable when using the *Sc* protein as a query for tBLASTn. Symbols on the tree represent opisthokonts (triangle), fungi (square), and Chytridiomycota (circle). Dashed lines surround chytrid species. *Ac*, *Acanthamoeba castellanii*; *Am*, *Allomyces macrogynus*; *An*, *Aspergillus nidulans*; *At*, *Arabidopsis thaliana*; *Bd*, *Batrachochytrium dendrobatidis*; *Ca*, *Candida albicans*; *Cr*, *Chalmydomonas reinhardtii*; *Dd*, *Dictyostelium discoideum*; *Hs*, *Homo sapiens*; *Mo*, *Magnaporthe oryzae*; *Nc*, *Neurospora crassa*; *Ng*, *Naegleria gruberi*; *Sc*, *Saccharomyces cerevisiae*; *Sp*, *Spizellomyces punctatus*; *Spo*, *Schizosaccharomyces pombe*; *Tb*, *Trypanosoma brucei*; *Tc*, *Trypanosoma cruzi*.

bioRxiv preprint doi: https://doi.org/10.1101/2025.05.13.653819; this version posted May 14, 2025. The copyright holder for this preprint (which was not certified by peer review) is the author/funder, who has granted bioRxiv a license to display the preprint in perpetuity. It is made available under aCC-BY 4.0 International license.

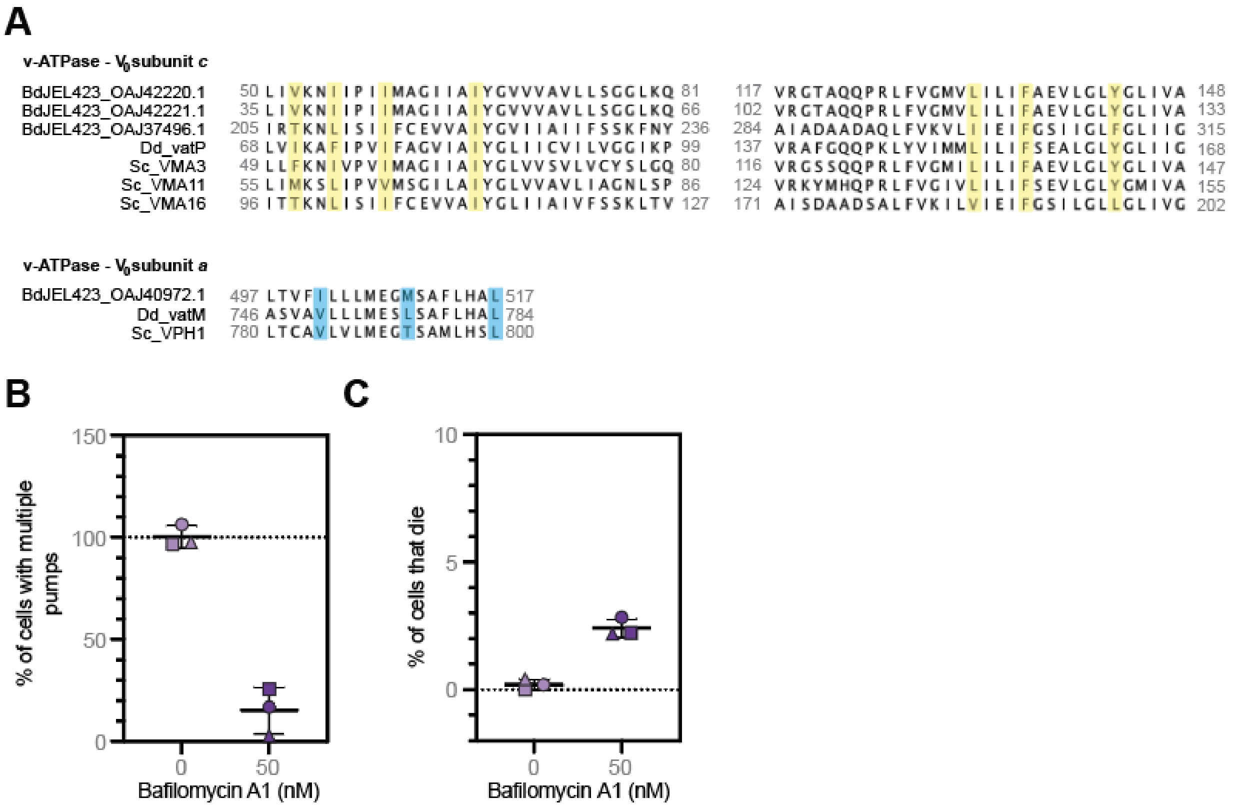

Figure S4. *Bd* is susceptible to the vacuolar ATPase inhibitor Bafilomycin A1. (**A**) TCoffee alignment of known and putative homologs for the indicated vacuolar ATPase subunits in the given species. Residues that form the bafilomycin A1 binding site (yellow) or that are disrupted by bafilomycin A1 binding (cyan) are highlighted. *Bd*, *Batrachochytrium dendrobatidis* strain JEL423; *Dd*, *Dictyostelium discoideum* AX4; *Sc*, *Saccharomyces cerevisiae* 288C. (**B**) Quantification of the percent of *Bd* zoospores that exhibit organelles undergoing multiple growing and shrinking cycles over a three minute period under agarose treated with 0 or 50 nM bafilomycin A1. Three independent biological replicates were performed, each represented by a shape. Mean and standard deviation of the three biological replicates are indicated by black lines. Two-tailed Student's t-test: p = 0.0007. **(C)** Quantification of the percent of *Bd* zoospores that die over a three minute period under agarose treated with 0 or 50 nM bafilomycin A1. Calculated by taking the difference between the percent of propidium iodide positive cells between the last and first frames of the time lapse. Three independent biological replicates were performed, each represented by a shape. Mean and standard deviation of the three biological replicates are indicated by black lines. Two-tailed Student's t-test: p = 0.0008.
